# Indirect Readout Drives Cre-*loxP* Recognition Thermodynamics

**DOI:** 10.1021/acs.biochem.6c00409

**Published:** 2026-10-22

**Authors:** Jonathan S. Montgomery, Megan E. Judson, Robert M. Bauer, Matthew J. Benedek, Caroline E. Hervey, Mark P. Foster

**Affiliations:** † The Ohio State Biochemistry Program, 2647 The Ohio State University, Columbus, Ohio 43210, United States of America; ‡ Department of Chemistry and Biochemistry, The Ohio State University, Columbus, Ohio 43210, United States of America; § Center for RNA Biology, The Ohio State University, Columbus, Ohio 43210, United States of America

## Abstract

Cre, a site-specific tyrosine DNA recombinase, enables targeted manipulation of genetic material without the production of cytotoxic double-strand DNA breaks. Though widely deployed in biotechnology, an incomplete understanding of how Cre recognizes its cognate *loxP* target limits its broader application in human health. Cre is highly specific for its cognate loxP sites, comprising an inverted pair of recombinase binding elements (RBEs) and an asymmetric spacer. Structural studies have shown that Cre protomers make few direct base-specific contacts to each RBE, suggesting that specificity arises through indirect readout, wherein noncognate sites are unable to adopt favorable binding-coupled conformations. We designed a set of *loxP* half-site variants predicted to alter local DNA shape characteristics, including minor-groove geometry and base-step parameters, and measured the thermodynamics of Cre binding by isothermal titration calorimetry. Variable-temperature thermograms and nuclear magnetic resonance spectra reveal that unfavorable mutations at noncontacted positions in the RBE reduce signatures of binding-coupled conformational changes. Moreover, mass photometry of Cre binding to oligonucleotides containing two properly spaced RBEs revealed that noncontacted positions in the intervening 8-base pair spacer influence cooperative dimerization of two Cre protomers at *loxP* sites and their synapsis to form catalytically active Cre_4_-*loxP*_2_ complexes. These results demonstrate that noncontacted positions contribute to specificity by encoding favorable DNA mechanics, offering new design principles for engineering Cre variants that target alternative DNA sequences.

## Graphical abstract

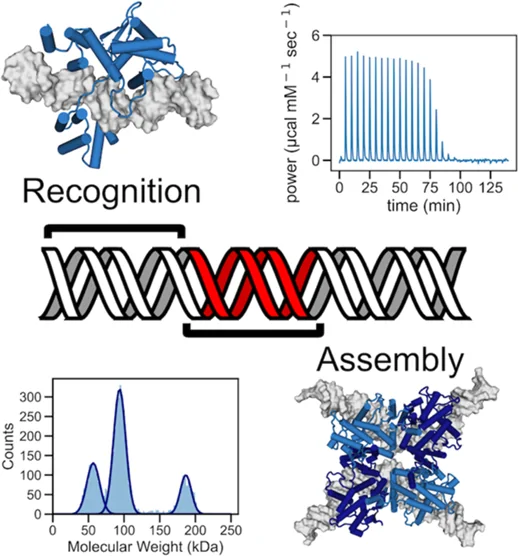

## Introduction

The ability to manipulate genetic material presents an opportunity to treat genetic diseases by excision and reintroduction of specific DNA sequences. Nuclease-based gene editing technologies such as CRISPR/Cas9 produce double-strand DNA (dsDNA) breaks at a site of interest that are repaired via mutagenic host repair pathways such as nonhomologous end joining.^1,2^ Tyrosine site-specific DNA recombinases, on the other hand, recombine DNA at paired homologous DNA sequences, catalyzing excision, inversion, or exchange of genetic material without producing dsDNA breaks.^3,4^ This capacity to preserve genome integrity makes tyrosine recombinases attractive tools for therapeutic gene editing.

Cre (Causes Recombination, UniProtKB entry P06956) is the most widely studied tyrosine recombinase.^3,5^ It has demonstrated utility in laboratory settings to produce conditional knockouts in transgenic mice^6^ and to introduce genes through recombinase-mediated cassette exchange.^7^ Cre is monomeric in solution and comprises two independently folding domains, an N-terminal core binding domain (CreCB) and a C-terminal catalytic domain (CreCat), that encircle the DNA forming a C-shaped clamp (Figure 1b,c).^8,9^ Cre recombines *loxP* sequences that contain two recombinase binding elements (RBEs) oriented as inverted repeats flanking an 8-base pair asymmetric spacer sequence (Figure 1a). Binding of two Cre monomers to the RBEs of a *loxP* site proceeds cooperatively, followed by synapsis of two Cre_2_-*loxP* dimers to form the tetrameric Cre_4_-*loxP*_2_ synaptic complex that is active for recombination.^9,10^

**Figure 1:**
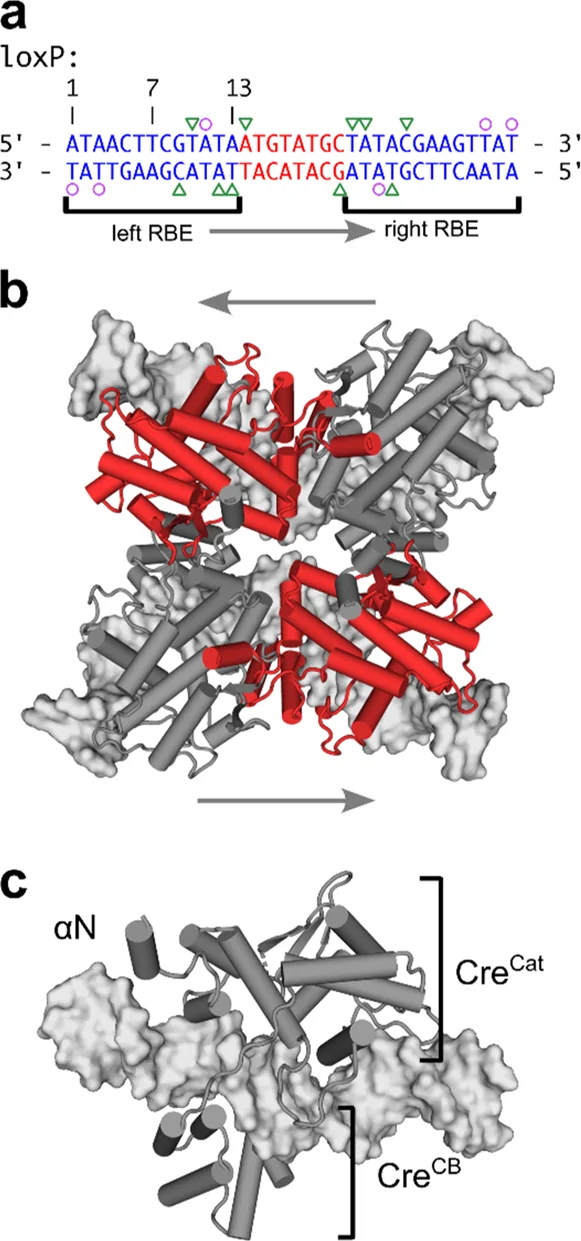
Assembly of Cre-*loxP*complexes is coincident with deformations in*loxP*DNA (a) *loxP* DNA sequence comprising two inverted RBEs (blue) flanking an asymmetric spacer (red). Triangles and circles denote direct major and minor groove contacts to DNA bases observed in high-resolution structures. Arrows indicate direction of the asymmetric spacer sequence. (b) Tetrameric Cre_4_-*loxP*_2_ synaptic complex (PDB 2HOI) with alternating protomers active for single strand DNA cleavage (red). (c) Cre bound as a monomer to a *loxP* half site (PDB 7RHY) features a ∼18° bend in the DNA.

While Cre has demonstrated utility in manipulating genomes, overcoming its obligate pairing with *loxP* sites would broaden its utility for gene editing. Despite high-resolution structures from X-ray crystallography of Cre bound in tetrameric structures with DNA,^3,11^ and multiple studies that evaluate the effect of DNA sequence on recombination,^12–14^ these have been of limited utility in guiding mutagenesis efforts to engineer Cre variants that effectively recombine alternative sites. The most successful approaches for altering Cre’s specificity have implemented substrate-linked directed evolution.^15–17^ However, these approaches are laborious and highly empirical and are yet to produce a general framework for understanding how DNA sequence recognition is achieved by Cre and its variants.^3^ Notably, because recombination is an ordered multistep process, protein and DNA mutations may affect multiple steps in the recombination pathway, including (1) the monomer-site recognition, (2) assembly of higher order oligomeric states, (3) DNA cleavage kinetics, and (4) direction of resolution of recombination intermediates.

With increased interest in retargeting Cre to biomedically relevant DNA sequences,^18,19^ understanding the basis of DNA site recognition by Cre has gained increased urgency. Broadly, the structural basis of sequence-specific DNA binding by proteins is described as arising from a combination of *direct* and *indirect* readouts. Direct readout features noncovalent interactions between specific amino acid side chains and nucleotide-specific chemical groups on the DNA bases, whereas indirect readout is achieved through sequence-specific differences in DNA shape.^20^ Structural and spectroscopic studies have found that recognition of a *loxP* half-site by Cre is coupled to distortions in the cognate DNA substrate that are not induced when binding noncognate sites.^9,21^ Given that Cre makes only three direct base-specific contacts to *loxP* half-sites (Figure 1),^9,10^ yet binds with thousand-fold specificity over noncognate DNA,^21^ this indicates that noncontacted positions play an essential role in defining Cre-*loxP* recognition.

In this report, we test the hypothesis that deleterious *loxP* mutations engineered to alter DNA shape while preserving direct protein–DNA contacts impair indirect readout by Cre. We employed isothermal titration calorimetry (ITC) and nuclear magnetic resonance (NMR) spectroscopy to characterize how noncontacted positions in the *loxP* sequence influence the binding thermodynamics and binding-coupled spectral signatures. We observed that mutations in the RBE that disfavor groove compression exhibit less favorable binding but paradoxically more favorable binding enthalpies. Protein NMR spectra revealed that mutations at noncontacted RBE positions abolish signatures associated with cognate DNA binding. Lastly, we found that mutations at noncontacted positions in the *loxP* spacer do not affect monomer-RBE binding but greatly perturb subsequent steps in assembly of Cre-*loxP* synaptic complexes. These results support a model in which noncontacted positions contribute to specificity by encoding favorable DNA mechanics, offering valuable design principles for engineering Cre variants to target alternative DNA sequences.

## Results

### Design of *loxP* Half Site Variants

To alter DNA shape while retaining direct protein–DNA contacts, we designed a set of *loxP* half site variants intended to disfavor the structural distortions from B-form geometry observed in structures of Cre-*loxP* complexes. AT-rich sequences, such as found in the *loxP* RBE, are associated with narrow minor grooves that favor arginine side chain contacts, while TpA steps are associated with local widening of the minor groove.^20^ NMR studies of model DNA duplexes have quantified flexibility metrics associated with the 10 possible base steps via the TRX (twist, roll, x-displacement) score,^22^ indicating that 5′-AT-3′ steps are rigid, while 5′-TA-3′ steps are the most flexible (Figure S1). Sequence-based DNA shape predictions using DEEP DNA Shape^23^ revealed a minor groove width pattern consistent with experimentally determined structures of Cre-*loxP* complexes^9,21^ and guided selection of half-site variants that perturb this pattern (Figure 2, Table S1).

**Figure 2:**
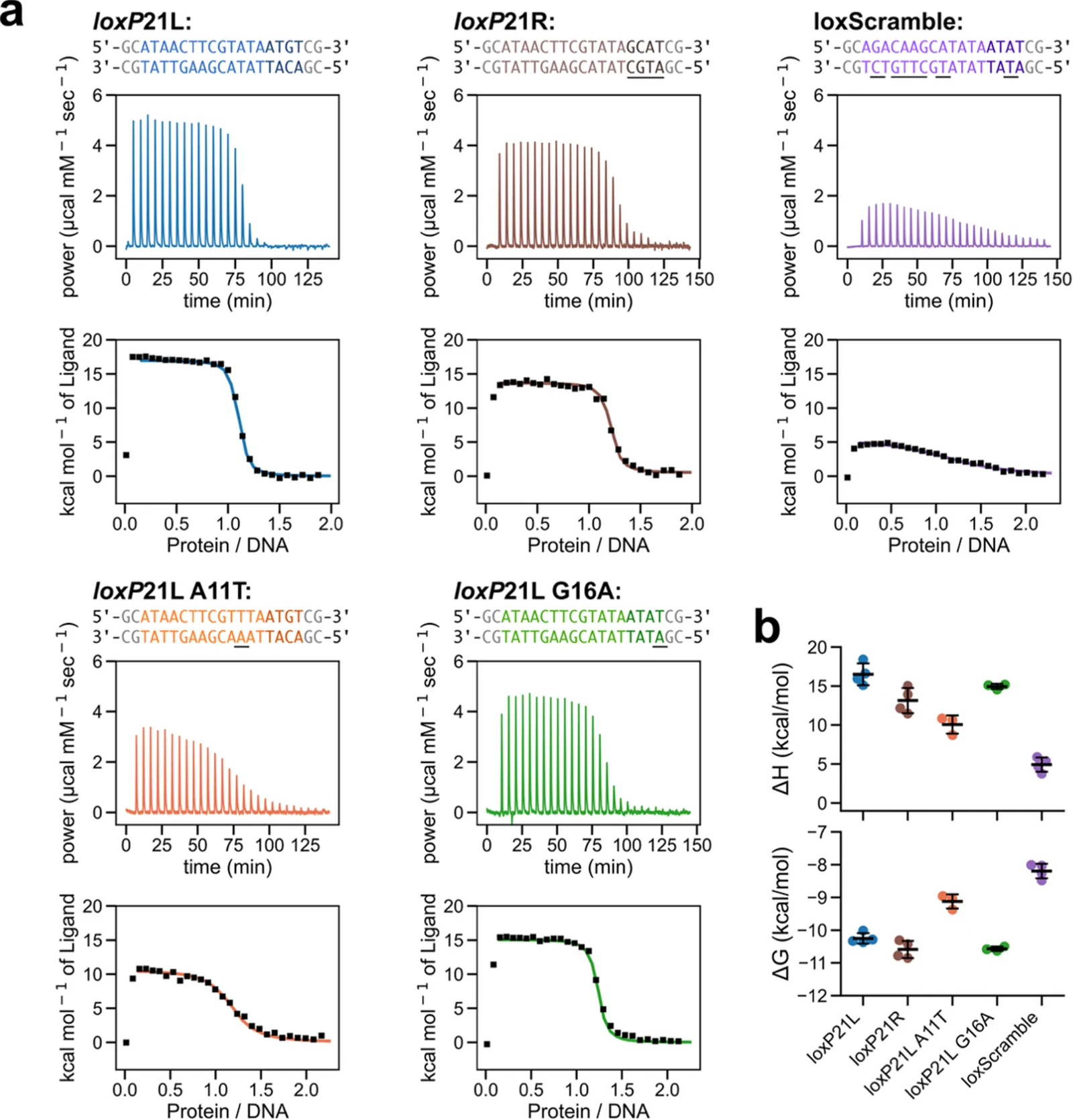
Noncontacted positions in the RBEs strongly influence half-site binding by Cre. (a) Baseline-corrected thermograms for titrations of Cre into each DNA substrate (top) and integrated heats fit to a single site binding model (bottom). Positions of mutations in *loxP* half-site variants are underlined. (b) Enthalpy (Δ*H*, top) and free energy of binding (Δ*G*, bottom) values obtained from fitting titrations to a single site binding model. Plots show values from 3 to 4 replicates and their mean and standard deviations (see Table S2). Compared to *loxP*21L (*K*_d_ = 17 ± 5 nM), Cre binding to *loxP*21R and G16A was statistically similar (*p*-values of 0.01 and 0.07, and 0.12 and 0.02, for Δ*H* and Δ*G*, respectively). Cre bound *loxP*21L A11T with lower affinity (*K*_d_ = 120 ± 40 nM) and less unfavorable binding enthalpy (*p*-values ≪0.01). Binding to loxScramble was even weaker affinity (*K*_d_ = 700 ± 200 nM) with further reduced positive enthalpy (*p*-values ≪ 0.01).

Two variants, A11T and G16A, introduce a rigid d*A*–d*T* base step at positions observed to undergo minor groove distortion upon Cre binding (Figure S1).^20,22–25^ Specifically, the A11T mutation converts a 5′-TAT-3′ stretch to 5′-TTT-3′, predicted to reduce minor groove flexibility and alter groove dimensions while preserving polar contacts between Arg282 of Cre and the Ade-N3 and Thy-O2 groups of the DNA.^9,10^ The loxScramble variant replaces all noncontacted positions with a sequence predicted to restore minor groove widths toward B-form DNA values.^23^ The *loxP*21L and *loxP*21R variants, which differ only in which half of the asymmetric spacer is included, serve as controls to test whether the spacer sequence affects RBE binding by Cre monomers.

### Thermodynamics of Recognition of *loxP* Half Site Mutants

We performed ITC experiments to quantify the effect of noncontact positions on the thermodynamics of DNA half-site binding by Cre. Solutions containing Cre were titrated into solutions containing one of the *loxP* half site variants. As observed with the native *loxP* half site,^21^ all titrations exhibited large positive (unfavorable) enthalpies (Δ*H*) of binding and thermograms were well described by a one-site binding model, with small variations from 1:1 stoichiometry attributed to uncertainties in component concentrations (Figure 2, Table S2). Integrated thermograms were fit to obtain the free energy, enthalpy, and entropy of Cre-half site binding.

We tested whether the spacer sequence affects thermodynamics of RBE recognition by Cre. Crystallographic and cryo-EM structures indicate that the first position of the spacer is involved in a nonspecific phosphate backbone contacts by Cre, and spacers symmetrized to match the left side of the asymmetric half-site have been reported to exhibit increased recombination efficiency relative to right symmetric spacers.^26^ To assess whether the spacer affects RBE binding, we performed ITC experiments of Cre binding to half-sites containing either the left or right spacer (*loxP*21L, *loxP*21R; Figure 2a). We previously found that titrations of Cre into *loxP*21L revealed a *K*_D_ of 17 ± 5 nM, Δ*H* of 17 ± 1 kcal mol^–1^, and *T*Δ*S* of 27 ± 1 kcal mol^–1^ at 15 °C.^21^ Fitting similar titrations for *loxP*21R yielded a *K*_D_ of 9 ± 4 nM, Δ*H* of 13.7 ± 0.7 kcal mol^–1^, and *T*Δ*S* of 24 ± 1 kcal mol^–1^ (Figure 2b, Table S2). These values are indistinguishable from those of binding to *loxP*21L, indicating that the large unfavorable enthalpy of half-site binding arises from interactions with the RBEs.

Mutations at noncontact positions that are designed to reduce DNA bending strongly impair recognition by Cre. Titrations with the A11T variant resulted in a weaker *K*_D_ of 120 ± 40 nM, a less positive Δ*H* of 10 ± 1 kcal mol^–1^, and corresponding *T*Δ*S* of 19 ± 1 kcal mol^–1^. Titrations for the G16A variant yielded values indistinguishable from the native *loxP* half-sites (*K*_D_ of 9 ± 1 nM, Δ*H* of 14.9 ± 0.4 kcal mol^–1^, and *T*Δ*S* of 25.5 ± 0.4 kcal mol^–1^). Titrations with the loxScramble variant, which features substitutions at noncontact positions and nucleotides that favor B-form helical structure, resulted in a much weaker *K*_D_ of 700 ± 200 nM, Δ*H* of 4.7 ± 0.8 kcal mol^–1^, and *T*Δ*S* of 12.9 ± 0.8 kcal mol^–1^. The results underscore the contributions of noncontact positions to the overall thermodynamics of Cre-DNA recognition.

We performed additional experiments to determine whether binding-coupled changes in ionization state contribute significantly to the experimentally observed positive binding enthalpy.^27–30^ If observed enthalpy changes include transfer of protons to or from the buffer, buffer-independent enthalpies can be obtained from a linear fit to Δ*H*_obs_ = Δ*H*_bind_ + *n*_H+_·Δ*H*_ion_, where Δ*H*_obs_ is the measured binding enthalpy, Δ*H*_bind_ is the buffer-corrected binding enthalpy, *n*_H+_ is the average number of protons transferred from buffer to the macromolecules, and Δ*H*_ion_ is the enthalpy of ionization of the buffer. We performed titrations of Cre into *loxP*21L in buffers with a range of ionization enthalpies (HEPES; 5.0 kcal mol^–1^, ACES; 7.3 kcal mol^–1^, and Tris; 11.4 kcal mol^–1^)^31^ (Figure S2, Table S3). This analysis yielded a buffer-corrected binding enthalpy of +14.9 kcal/mol, with 0.6 ± 0.3 ionization events at pH 7.0. These results indicate that protonation is not a major contributor to the enthalpy of Cre-RBE binding, supporting the conclusion that binding-coupled conformational changes are the primary source of the observed positive Δ*H*.

### Thermodynamic Signature of Cognate Cre-*loxP* Recognition from Temperature-Dependent Binding Enthalpy

A negative heat capacity change, Δ*C*_p_, exceeding that predicted from burial of solvent-accessible surface area at the protein–DNA interface provides a thermodynamic signature of binding-coupled conformational changes during specific protein–DNA recognition.^29,32,33^ We quantified this thermodynamic parameter from the linear relation ${\Delta C}_{p}=\frac{d\Delta H}{dT}$ by recording ITC data over a range of temperatures from 283 to 298 K (Figure 3, Table S4). Binding between Cre and the native *loxP*21L yielded a large negative Δ*C*_p_ (−830 ± 90 cal mol^–1^ K^–1^), whereas for each variant, a much smaller value was obtained (*loxP*21L A11T, Δ*C*_p_ = −260 ± 70 cal mol^–1^ K^–1^; loxScramble, Δ*C*_p_ = −120 ± 60 cal mol^–1^ K^–1^). Binding free energies, Δ*G*, were relatively insensitive to temperature over this temperature range (Figure 3b).

**Figure 3:**
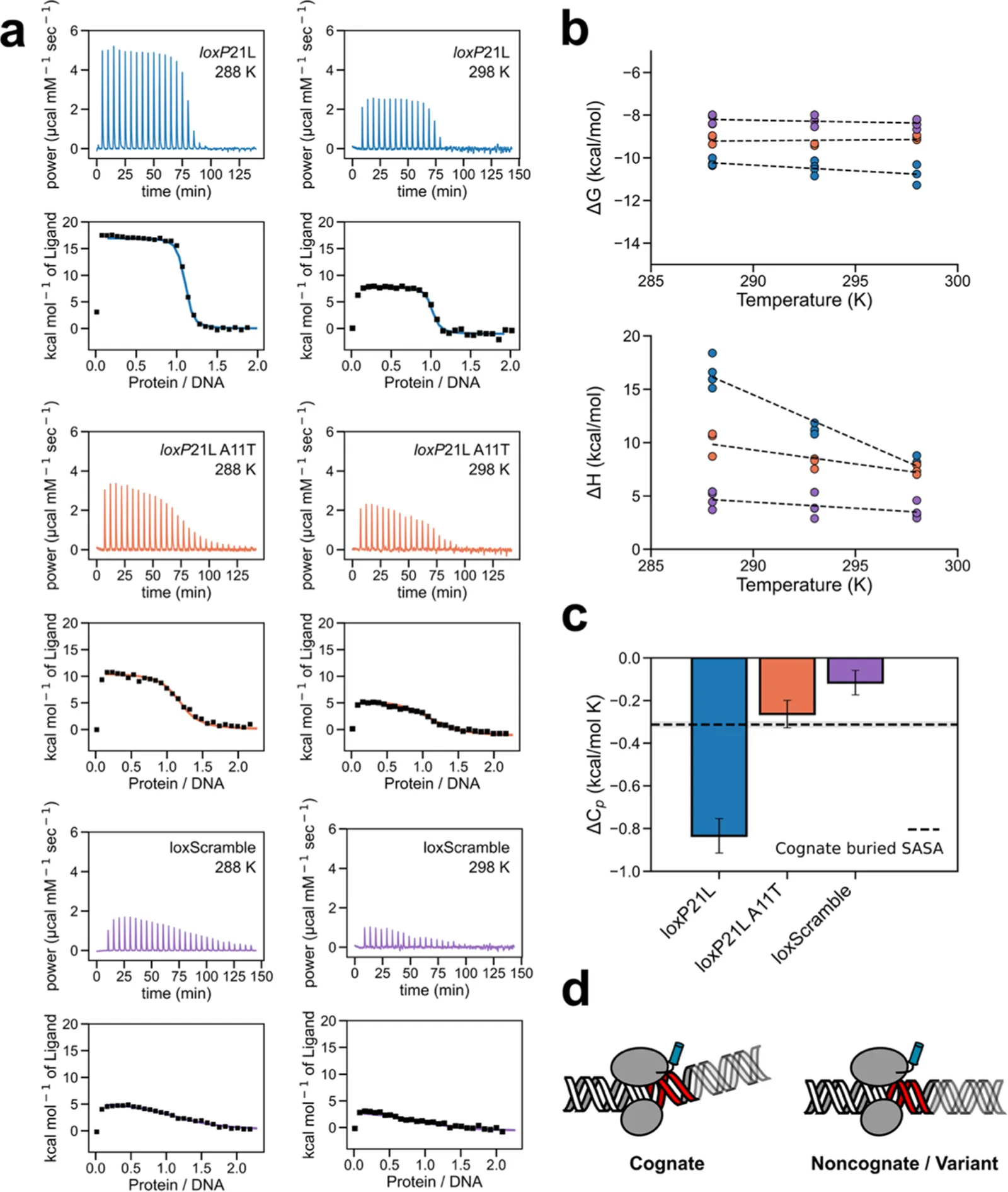
Noncontact positions in the*loxP* half-site define thermodynamic signature of specific recognition. (a) Representative thermograms for *loxP*21L (top), *loxP*21L A11T (middle), and loxScramble (bottom) reveal temperature-dependent binding enthalpy. (b) Resulting thermodynamic parameters from fitting titrations to single site binding models. The points represent fitted values from 3 to 4 individual replicates. (Top) The binding free energy (Δ*G*) for each oligo is relatively temperature-independent, while (bottom) the temperature-dependent binding enthalpies (Δ*H*) are quite distinct. (c) Δ*C*_p_ obtained from a linear fit of the temperature dependence of Δ*H*. Uncertainties were estimated from the standard error of the fit. The horizontal dashed line is the Δ*C*_p_ predicted from buried surface area in PDB 7RHY. (d) Model for coupled DNA bending and large negative Δ*C*_p_ for specific binding to cognate DNA, and smaller value for noncognate oligos.

The contribution to Δ*C*_p_ from changes in solvation was estimated from analysis of surface area burial in the cryo-EM model of monomeric Cre bound to a *loxP* half site (PDB 7RHY^9^). Changes in solvation upon burial of accessible polar and nonpolar surfaces is a major contributor to the thermodynamics of protein folding, and of the interactions of proteins with nucleic acid ligands.^27,29,32,33^ Empirically determined proportionality constants allow contributions to Δ*C*_p_ to be estimated from changes in polar and nonpolar accessible surface area: Δ*C*_p_ = (0.32 ± 0.04 kcal mol^–1^ K^–1^)·Δ*A*_np_ – (0.14 ± 0.04 kcal mol^–1^ K^–1^)·Δ*A*_p_.^32^ Contact surface area calculations for the Cre-half-site complex reveal burial of 4371 Å^2^ of solvent-exposed surface area, with 54% polar and 46% nonpolar surface area,^34^ yielding an estimate of −310 ± 10 cal mol^–1^ K^–1^ from solvent exclusion in the protein–DNA interface. This value is much smaller than observed for binding of Cre to *loxP*21L, within error of the value for *loxP*21L A11T, and greater than that observed for loxScramble (Figure 3c). For the cognate *loxP*21L interaction with Cre, the excess ∼500 cal mol^–1^ K^–1^ is consistent with the coupling of substantial structural rearrangements to site recognition. The much smaller negative Δ*C*_p_ values obtained for the A11T and Scramble variants suggest that these lower affinity interactions are not coupled to sizable conformational changes and/or or do not bury as much surface area upon binding.

### NMR Reveals Characteristic Spectral Signatures of Protein Conformational Changes Coupled to *loxP* Recognition

To evaluate whether the unique thermodynamic signatures of Cre binding to the cognate and variant half-sites could be attributed to protein conformational changes, we compared NMR spectra of [U–^15^N]-Cre bound to *loxP*21L with those bound to *loxP*21L A11T and loxScramble half-sites (Figures 4 and S3). NMR spectra of Cre mixed with excess DNA substrate result in broadened peaks relative to the apo protein, indicative of the slower tumbling complex in solution (Figure S4). The spectrum of Cre bound to *loxP*21L A11T overlays well with that of Cre bound to the cognate *loxP*21L, whereas the Cre bound to loxScramble is quite different, resembling that of Cre bound to a noncognate DNA substrate (NCD1) (Figures 4, S3). Spectral similarities were quantified using a nearest-neighbor distance score, which reciprocally measures the median difference in the chemical shift for signals in a test spectrum with the signals in a corresponding reference spectrum, wherein lower scores indicate similarity. In addition, several well-resolved resonances in the catalytic domain, assigned by comparison to the apo protein spectrum and distal from the DNA interface, report on structural differences between the various Cre-DNA complexes (Figure 4c,d).

**Figure 4:**
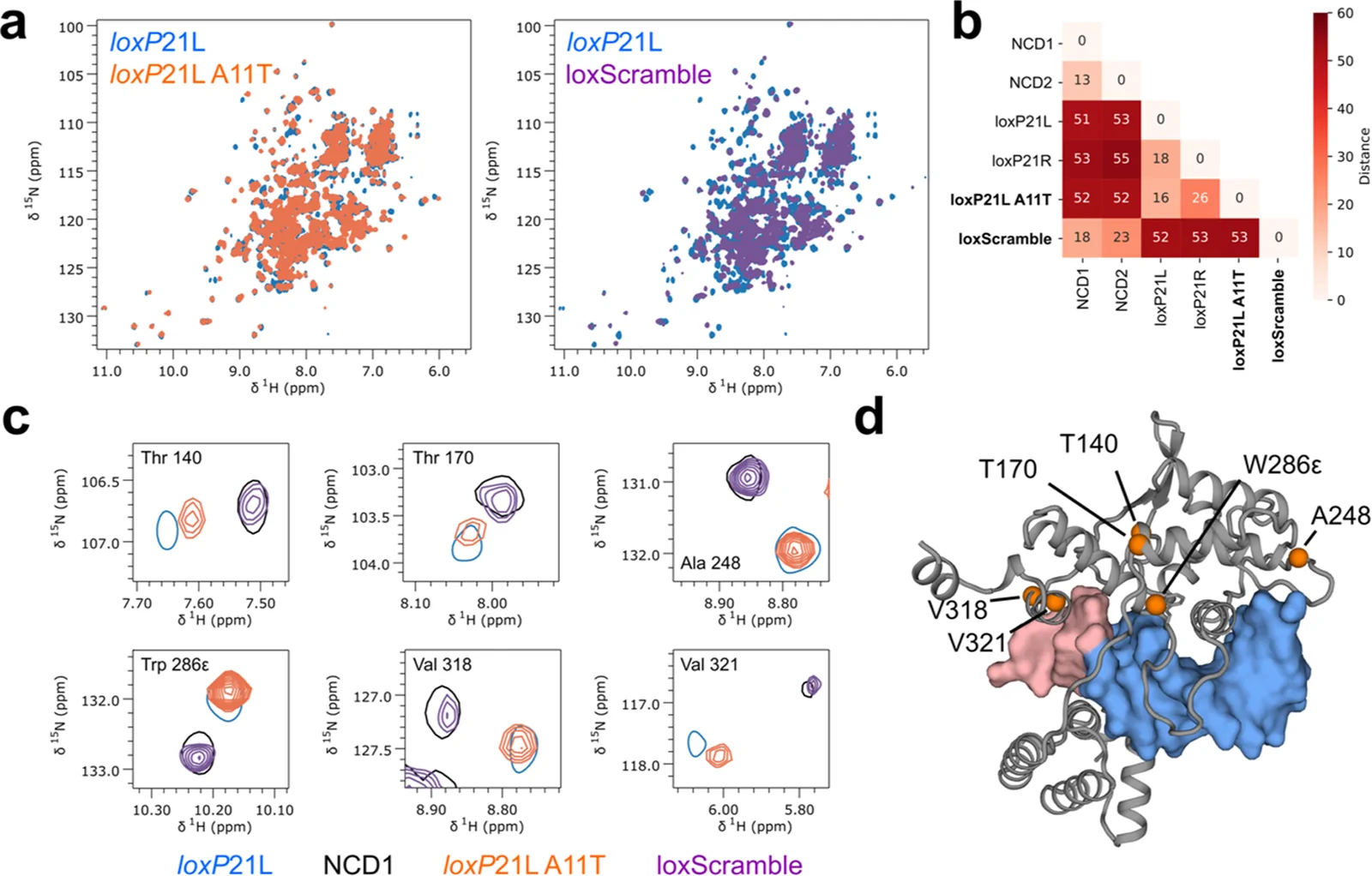
NMR spectra reveal structural role for noncontact positions in recognition. (a) Overlay of TROSY-HSQC spectra of [U–^15^N]-Cre bound to *loxP*21L (blue), or its variants A11T (orange) and loxScramble (purple). (b) Pairwise spectral similarities expressed as the median difference in positions between overlaid NMR signals in parts per billion (ppb). (c) Subsets of 2D spectra of Cre bound to *loxP*21L (blue, single contour), *loxP*21L A11T (orange), loxScramble (purple), and to noncognate DNA (black, single contour). Representative signals were selected because they are well-resolved, assigned in free Cre, distributed throughout the protein, and not in the DNA interface. (d) Model of Cre bound to a cognate half site (chain A, 2HOI)^10^ with residues in panel (c) shown as orange spheres; the RBE is blue and spacer nucleotides are red. Spectra reveal similar structures for Cre bound to *loxP*21L and A11T, whereas Cre bound to the half-site mutated at all noncontact positions, loxScramble, resembles that bound to a random sequence, NCD1.

NMR spectra reveal that the structural ensemble of Cre is sensitive to sequence identity at noncontacted RBE positions, distinguishing cognate from noncognate binding modes. Qualitatively and quantitatively, the spectra of Cre bound to two noncognate DNA substrates with no similarity to the *loxP* RBE (NCD1 and NCD2)^35^ are highly similar (median of 13 ppb), as are the spectra of Cre bound to left- and right-half sites (median of 18 ppb). Cre bound to *loxP*21L A11T exhibits spectral signatures similar to those of Cre bound to *loxP*21L and *loxP*21R (median CSP values of 16 and 26 ppb, respectively). Conversely, when bound to loxScramble, the bound conformation of Cre shifts toward that observed for noncognate DNA substrates (median CSPs of 18 and 23 ppb, compared to 52 and 53 ppb for cognate sequences). These spectra underscore the importance of noncontacted RBE positions as important structural determinants of Cre-*loxP* half-site recognition.

### Mutations in the *loxP* Spacer Perturb Synaptic Assembly

We used mass photometry to assess the effect of spacer nucleotides on the dimerization and synaptic assembly. While the measured thermodynamics of monomeric Cre binding to *loxP*21L G16A exhibited no significant difference from the WT *loxP*21L sequence (Figure 2), recombination assays show the point mutation results in a 50% reduction in the recombination efficiency.^13^ Similarly, prior studies showed that a *loxP* sequence containing a spacer symmetrized to the right half (*loxSR*) exhibits reduced recombination efficiency^26^ and weakened synapsis,^10^ despite Cre retaining high-affinity binding to half-site oligonucleotides containing that sequence (Figure 2). The observations that these sequences matter in the context of recombination suggest that the mutations affect steps after RBE recognition, such as dimerization and synapsis.

Distributions of Cre-DNA complexes detected by mass photometry revealed a strong influence of spacer sequence on assembly of synaptic complexes. The WT *loxP* sequence, *loxP* G16A, and *loxSR* (Table S1) were mixed with the catalytically inactive but synapsis-competent variant, Cre K201A,^10^ at various concentrations to observe changes in oligomeric distributions. At DNA concentrations above the expected 14 nM K_D_ of synapsis^10^ (50 nM DNA, 100 nM Cre K201A, Figure 5 a), we observe three populations indicative of monomeric, dimeric, and tetrameric Cre-*loxP* complexes. These same three states are observed with *loxP* G16A, but the relative population of the tetrameric complex is decreased (Figure 5b). For *loxSR*, only states corresponding to the monomeric and dimeric complexes were observed. Experiments at lower concentrations were used to assign the identity of the states (Figure S5). These results corroborate prior findings and suggest that mutations in the spacer that reduce recombination efficiency do so, at least in part, by destabilizing the active tetrameric complex.

**Figure 5:**
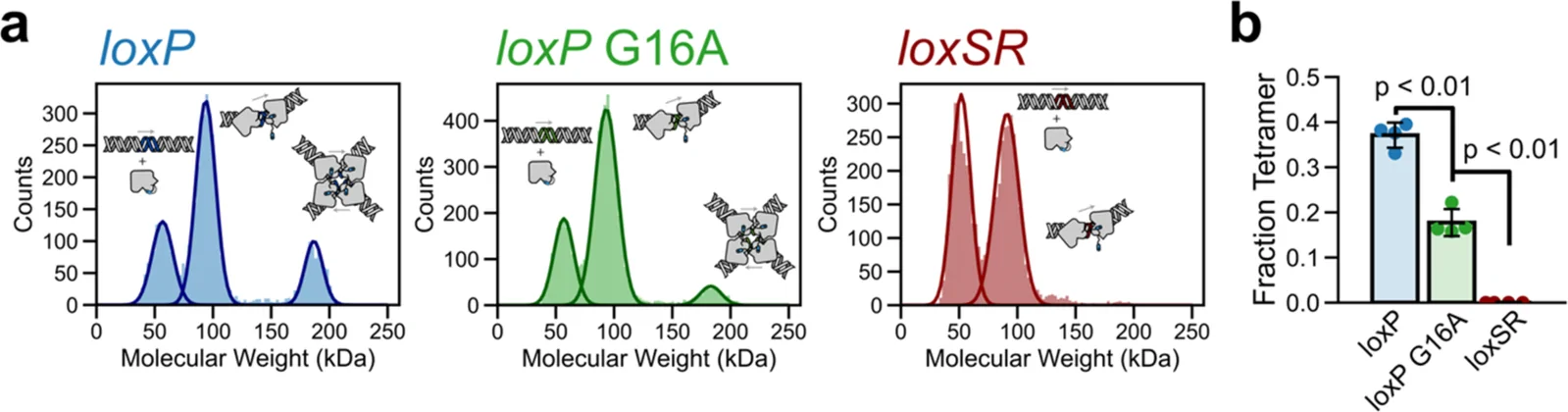
Noncontacted positions in the*loxP* spacer affect dimerization and synapsis of Cre-*loxP* complexes. (a) Population distributions of *Cre-loxP* complexes were measured using mass photometry using catalytically inactive Cre-K201A and DNA oligonucleotides comprising two RBEs and an 8-bp spacer. Complexes of Cre-*loxP* (blue), Cre-*loxP* G16A (green), and Cre-*loxSR* (red) were formed by mixing components at 1:1 Cre to RBE ratios. Resulting histograms were fit with a Gaussian mixture model to estimate relative populations of each oligomeric state (indicated schematically) and corrected for mass-dependent differences in particle diffusion. (b) Bar graphs represent the mean and standard deviation of the tetrameric fraction for each DNA substrate with points indicating each replicate. Significance was estimated using an unpaired *t*-test. Cre readily formed tetramers with *loxP*, while those were disfavored by the G16A spacer mutation, and not observed at all with the right-side symmetrized spacer *loxSR*.

## Discussion

In this work, we assessed the role of DNA shape and flexibility in site recognition and assembly of higher order Cre-*loxP* complexes. We used sequence-based predictions of DNA structure and base-step flexibility to design *loxP* half-site variants that preserved direct contacts but were designed to resist protein-induced conformational changes. Titration calorimetry revealed that Cre binds these stiffer half-site substrates with reduced affinity but more favorable binding enthalpy. The unfavorable Δ*H* and large negative Δ*C*_p_ observed for cognate but not to variant half-site binding are attributable to energetically costly deformations upon complex formation. NMR spectra of the protein bound to these DNA half-sites revealed that despite preserving direct contacts, alteration of noncontacted positions shifts the bound conformation toward the conformation observed when bound to noncognate DNA. Additionally, we found that a single point mutation (G16A) in the *loxP* spacer, previously shown to affect the recombination efficiency, destabilized higher order assemblies without affecting half-site recognition. These observations establish that noncontacted positions contribute to Cre-*loxP* specificity through DNA mechanical properties, with distinct roles in half-site recognition and higher order assembly.

### Favorable DNA Deformations Contribute to Specificity

High-resolution structures of Cre-*loxP* complexes indicate that Cre induces minor groove compression and DNA bending at the TATA track in cognate *loxP* half-site DNA.^3,9,11,36^ Site-specific high-affinity binding to *loxP* features a large unfavorable binding enthalpy (Δ*H*) characteristic of the energetic cost of DNA deformation upon binding.^21,37–39^ To better characterize binding-coupled conformational changes in *loxP* half-site variants, we quantified Δ*C*_p_ from the temperature dependence of Δ*H*.^29,32^ For the cognate *loxP*21L half-site, the measured Δ*C*_p_ was much more negative than predicted based on a rigid body association model based on buried surface area, consistent with coupled conformational changes.^9^ In contrast, the measured Δ*C*_p_ for *loxP*21L A11T is not more negative than predicted from buried surface area, suggesting that DNA deformations are not coupled to binding this variant. The A11T mutation disrupts the TATA track, removing the flexible TpA base steps and presumably preventing DNA bending necessary for high-affinity recognition. The much smaller negative Δ*C*_p_ for Cre binding to loxScramble suggests that its resistance to deformation results in reduced structural complementarity at the binding interface, despite retaining the nucleotides involved in direct readout.

### Protein Conformational Changes Are Coupled to Recognition

Given its flexibly linked DNA binding and catalytic domains, Cre is expected to undergo conformational changes upon forming a C-clamp around a *loxP* RBE. NMR evidence indicates that the C-terminus of Cre adopts an autoinhibited conformation that occludes the DNA-binding surface of the catalytic domain, yet is extended upon cognate *loxP* binding.^8,35^ This C-terminal region forms a helix (αN) implicated in promoting assembly of higher order Cre-*loxP* complexes and activating Cre protomers for strand cleavage.^9,10^ Congruence in protein amide NMR spectra between cognate *loxP*21L and the A11T variant suggests that in both complexes, the ensemble-averaged structure shifts toward the extended conformation (Figure 4), despite >10-fold difference in affinity and contrasting Δ*C*_p_ values (Figures 2 and 3). This suggests that the excess negative Δ*C*_p_ observed in cognate binding is a result of DNA deformations rather than binding-coupled protein folding. In contrast, despite retaining positions involved in direct readout, the spectrum of Cre in the complex with the loxScramble half-site resembles that of a nonspecific complex (Figure 4). These observations are consistent with a model in which high-affinity cognate binding is coupled to unlocking the autoinhibited conformation, with implications for assembly of higher-order complexes.

### Noncontacted Positions in the Spacer Perturb Dimerization and Synapsis

Experiments with longer DNA substrates containing intact *loxP* sites revealed the importance of indirect readout in the assembly of higher-order recombinogenic Cre-*loxP* complexes. The specificity of Cre recombinase is not only predicated on the ability of a monomer to recognize the cognate RBEs but also on the cooperative assembly of active Cre_4_-*loxP*_2_ complexes mediated by protein–protein interactions across bent DNA duplexes.^9,10,40^ While half-site substrates containing the *loxP* G16A and loxSR mutations are recognized by Cre with thermodynamics equivalent to those of the cognate sequences, full-length *loxP* substrates with these mutations in the spacer region exhibit reduced recombination.^26^ Our mass photometry experiments showed that a single rigidifying mutation in the *loxP* spacer (G16A) significantly reduces the population of Cre_4_-*loxP*_2_ complexes, while a symmetric right spacer abolishes synapsis of Cre_2_-*loxP* complexes under the tested conditions, consistent with their reported effects on recombination.^13^ These results underscore the importance of noncontacted positions in the *loxP* spacer in defining the stability of synaptic complexes and subsequent recombination.

### Stepwise Model of Site Selection

Thermodynamic and spectroscopic data support a stepwise model of site selection by Cre. Upon encountering a candidate binding site, DNA shape may serve as an initial selectivity checkpoint, with favorable groove geometry permitting the formation of base-specific contacts that trigger conformational changes in the protein. Further structural accommodation dependent on intrinsic DNA flexibility, coupled with protein conformational changes, results in a high-affinity, conformationally coupled complex. In aggregate, these structural deformations culminate in DNA bending that enables subsequent Cre monomers to assemble cooperatively by enabling protein–protein interactions required for assembly and ordered cleavage of a tetrameric synaptic complex.

## Conclusions

These experiments highlight the role of DNA flexibility in the indirect readout and specificity of Cre-*loxP* recognition. Detailed calorimetric analysis revealed that direct base-specific contacts to the *loxP* sequence do not make dominant contributions to high-affinity binding, rather Cre recognizes target *loxP* half-sites through coupled conformational changes in the form of DNA deformations. We show that noncontacted positions in the *loxP* sequence drive site-specific recognition and that variants with altered RBEs exhibit thermodynamic and spectral signatures comparable to noncognate DNA. We also observe that mutations in the *loxP* spacer can reduce the recombination efficiency by destabilizing the synaptic complex rather than perturbing monomer recognition. The thermodynamic and spectroscopic signatures of site recognition identified here provide a framework for engineering Cre variants with altered DNA site specificities.

## Methods

### Protein Expression and Purification

A pET21A plasmid encoding for the wild-type Cre enzyme (UniProt ID P06956) was provided by Gregory Van Duyne (University of Pennsylvania Philadelphia, PA). The plasmid encoding Cre K201A was described previously.^9,35^ Electrocompetent *Escherichia coli* T7 express LysY/Iq cells (New England Biolabs) were transformed using a 1 mm cuvette and plated on LB agarose plates supplemented with 0.1 mg/mL carbenicillin for antibiotic selection and grown overnight at 37 °C. A single colony was picked from the antibiotic selection plate and was used to inoculate to a liquid 50 mL LB media culture supplemented with 0.1 mg/mL carbenicillin. This culture was grown overnight at 37 °C shaken at 220 rpm. Following overnight growth, 10 mL of liquid culture was transferred to 1 L of either rich LB media or M9 minimal media^35^ supplemented with 1 g/L ^15^NH_4_ as the sole nitrogen source for protein overexpression. Liquid cultures were additionally supplemented with 0.1 mg/mL carbenicillin for antibiotic selection and incubated at 37 °C shaking at 220 rpm. Once the optical density at 600 nm reached 0.6–0.8, 400 μL of 1 M IPTG (Isopropyl β-D-1-thiogalactopyranoside) was added to induce protein expression and the cultures were allowed to incubate for an additional 4 h. Following the induction period, cells were pelleted by centrifugation at 4225*g*, liquid media was decanted, and pellets were frozen at −80 °C.

Frozen cell pellets containing expressed proteins were thawed on ice and resuspended in 35 mL of buffer A (40 mM Tris pH 7.0, 100 mM NaCl) supplemented with 1 mM EDTA and one tablet of miniComplete EDTA free protease inhibitor (Roche). Cells were lysed by sonication on ice (Qsonica, two cycles of 50% amplitude for 5 min, 5 s on and 5 s off), and the insoluble fraction was pelleted by centrifugation at 26891*g* for 45 min at 4 °C. The supernatant was syringe filtered using a 0.2 μm filter (Pall) and first purified using cation exchange chromatography using a 5 mL SPFF column (Cytiva) eluted with a linear salt gradient (60 mL, 40 mM Tris pH 7.0, 100 mM to 1 M NaCl). Fractions containing Cre protein were isolated and diluted 1:4 in buffer A to reduce the ionic strength and were further purified by affinity chromatography with a 5 mL heparin column (Cytiva) eluted with a linear salt gradient (90 mL, 40 mM Tris pH 7.0, 100 mM to 1.5 M NaCl). The eluted protein was concentrated to 2 mL before loading onto size exclusion using a 120 mL S75pg column (Cytiva) in S75 buffer (40 mM Tris pH 7.0, 500 mM NaCl, 5 mM DTT). Purity was ensured to be >95% using an SDS-PAGE gel.

### DNA Design and Purification

Variant *loxP* half-site sequences were first designed by predicting the effect of mutations at noncontacted positions on the DNA minor groove width, using DeepDNAShape (https://deepdnashape.usc.edu).^23^ Sequences were chosen that perturb the predicted minor groove expansion and compression between positions 12–18 (Figures 1a, S1), as expansion and compression at these positions is also observed in the structure of Cre bound to a *loxP* half-site. The resulting sequences were then compared to their TRX (twist, roll, *x*-displacement) score^22^ to estimate the effect of the mutation on predicted DNA flexibility.

Single-strand DNA oligos (Integrated DNA Technologies) were resuspended in miliQ H_2_O to 100 μM, and concentrations were confirmed by nanodrop (Thermo Scientific). Resuspended top and bottom strand oligos were combined in equimolar amounts in annealing buffer (10 mM Tris pH 7.0 and 100 mM NaCl) and heated at 95 °C for 10 min. The water bath was then turned off, and the oligos slowly cooled overnight to allow for proper annealing. Annealed DNA duplexes were purified to remove ssDNA contamination by anion exchange chromatography using a QHP column (Cytiva) with a linear buffer gradient (10 mM Tris pH 7.0, 25 mM to 1 M NaCl). Purified dsDNA was ensured by analyzing chromatography fractions on a 2% (w/v) agarose gel impregnated with Sybr Safe dye (Invitrogen) for visualization.

### ITC

WT Cre and DNA substrates were dialyzed overnight at 4 °C into 1 L of ITC buffer (20 mM HEPES pH 7.0, 250 mM NaCl) using either 6–8 kDa MWCO dialysis tubing (Spectrum Laboratories) or 3.5 kDa MWCO dialysis cassettes (Thermo Scientific) in the same container to limit heats of dilution. Protein and DNA concentrations were measured after dialysis by measuring absorbance at 280 and 260 nm, respectively (ε_280,WT_ = 48,930 M^–1^ cm^–1^, ε_260,loxP21L_ = 333,780 M^–1^ cm^–1^, ε_260,loxP21R_ = 334,427 M^–1^ cm^–1^, ε_260,loxP21L A11T_ = 330,581M^–1^ cm^–1^, ε_260,loxP21L G16A_ = 341,315 M^–1^ cm^–1^, and ε_260,loxScramble_ = 341,074 M^–1^ cm^–1^). Protein was loaded into the syringe at concentrations ranging from 30 to 150 μM, and DNA was loaded into the cell at concentrations ranging from 3 to 15 μM. ITC experiments were run using a VP-ITC (Malvern Panalytical) at temperatures ranging from 15 to 25 °C, reference power of 5 μcal/s. Injection volumes were set to 10 μL with an interinjection delay of 300 s and a stirring speed of 307 rpm. Titrations of each *loxP* variant were performed with 3–4 replicates. Baselining and integration of the thermograms was completed using NITPIC,^41^ and titrations were fit using ITCSIMLIB^42^ to a single-site binding model and fit parameters and uncertainties for each titration were determined by bootstrapping with 1000 iterations. Changes in polar and nonpolar solvent accessible surface area were computed for the structure of monomer Cre bound to a *loxP* half site substrate (PDB 7RHY) using dr-SASA (github.com/nioroso-x3/dr_sasa_n).^34^

### NMR

Protein–DNA complexes were formed by mixing [U–^15^N]-Cre with the 2-fold excess DNA substrate in high salt NMR buffer (20 mM HEPES pH 7.0, 500 mM NaCl, 5 mM DTT). The mixture was then dialyzed stepwise at 4 °C, each step a 100-fold dilution for 8 h into NMR buffer with reduced ionic strength (500 → 200 → 100 → 25 mM NaCl) in 6–8 kDa MWCO dialysis tubing (Spectrum Laboratories). Complexes were then concentrated by centrifugation using a 3 kDa spin cutoff filter to an approximate final concentration of 300 mM Cre. 250 μL of the complex were supplemented with 10% (v/v) D_2_O and 225 μM DSS (2,2-dimethyl-2-silapentane-5-sulfonate) for spin locking and chemical shift referencing, respectively, in 3 mm NMR tubes. ^1^H–^15^N TROSY-HSQC spectra were recorded using a Bruker AVANCE 800 MHz spectrometer equipped with a triple-resonance inverse (TXI) cryoprobe. Acquisition parameters for each TROSY-HSQC spectrum are listed in Table S5. Data were processed using NMRFx Analyst^43^ (nmrfx.org). Spectral similarity scores were computed from

$$distance\left(ppb\right)={\left[{\left({\Delta \delta }^{1}H\right)}^{2}+{\left(\frac{\Delta \delta^{15}N}{5}\right)}^{2}\right]}^{1/2}\cdot 1000$$

### Mass Photometry

Purified Cre K201A, *loxP*38, *loxP*38 G16A, and *loxSR* were dialyzed overnight against buffer containing 20 mM HEPES pH 7.0 and 100 mM NaCl for a 3:1000 dilution. Protein–DNA complexes were allowed to equilibrate for more than 2 h before experimentation. Borosilicate slides were cleaned in triplicate with alternating washes of miliQ-H_2_O and isopropanol, finishing with H_2_O before drying with nitrogen gas. Mass photometry measurements were completed using a Refeyn TwoMP instrument with buffer-free focusing and direct loading of samples for measurement. Calibration of radiometric contrast to molecular weights was completed using standards with the known molecular weights of 86, 172, 258, and 344 kDa from the Refyn MassFerence P1 calibrant diluted in the buffer above. Movies measuring radiometric contrast of scattering events were recorded for 60 s, and contrast values were converted to molecular weights using the calibration curve. Data were exported as individual molecular weight values, and histograms were fit using a Gaussian mixture model using the SciPy and Scikit-learn python libraries. Estimated populations were calculated from the relative weight of the Gaussian fit, normalized to molecular weight-dependent differences in diffusion for each species (*D* ∝ MW^–1/3^).^44^ Code is available at https://github.com/mpfosterlab. Fraction of Cre monomers that assemble into tetrameric complexes was estimated as

$$F_{tetramer}=\frac{4^{*}P_{tetramer}}{4^{*}P_{tetramer}+{3^{*}P}_{trimer}+{2^{*}P}_{dimer}+P_{monomer}}$$

## Supplementary Material



## Notes

Sequence-dependent structure and flexibility predictions; buffer ionization enthalpy effect on observed binding enthalpy; NMR spectra of ^15^N-Cre alone and bound to DNA sequence variants; mass photometry data histograms of Cre-DNA complexes; DNA oligonucleotide sequences; individual ITC experiment results; and NMR data acquisition parameters (PDF)
