# Supplementary material for: Indirect Readout Drives Cre-loxP Recognition Thermodynamics
Source: Biochemistry. 2026 Oct 22;65(21):3399–408. doi: 10.1021/acs.biochem.6c00409 (PMC13738788; doi:10.1021/acs.biochem.6c00409)
Supplement: Supplementary file 1 [file bi6c00409_si_001.pdf]

Indirect Readout Drives Cre-*loxP* Recognition Thermodynamics

Jonathan S. Montgomery[1,2], Megan E. Judson[2], Robert M. Bauer[2], Matthew J. Benedek[1,2], Caroline E. Hervey[2], Mark P. Foster[1,2,3*].

[1]The Ohio State Biochemistry Program, The Ohio State University, Columbus, 43210, United States of America

[2]Department of Chemistry and Biochemistry, The Ohio State University, Columbus, 43210, United States of America

[3]Center for RNA Biology, The Ohio State University, Columbus 43210, United States of America

Corresponding Author: Mark P. Foster (foster.281@osu.edu)

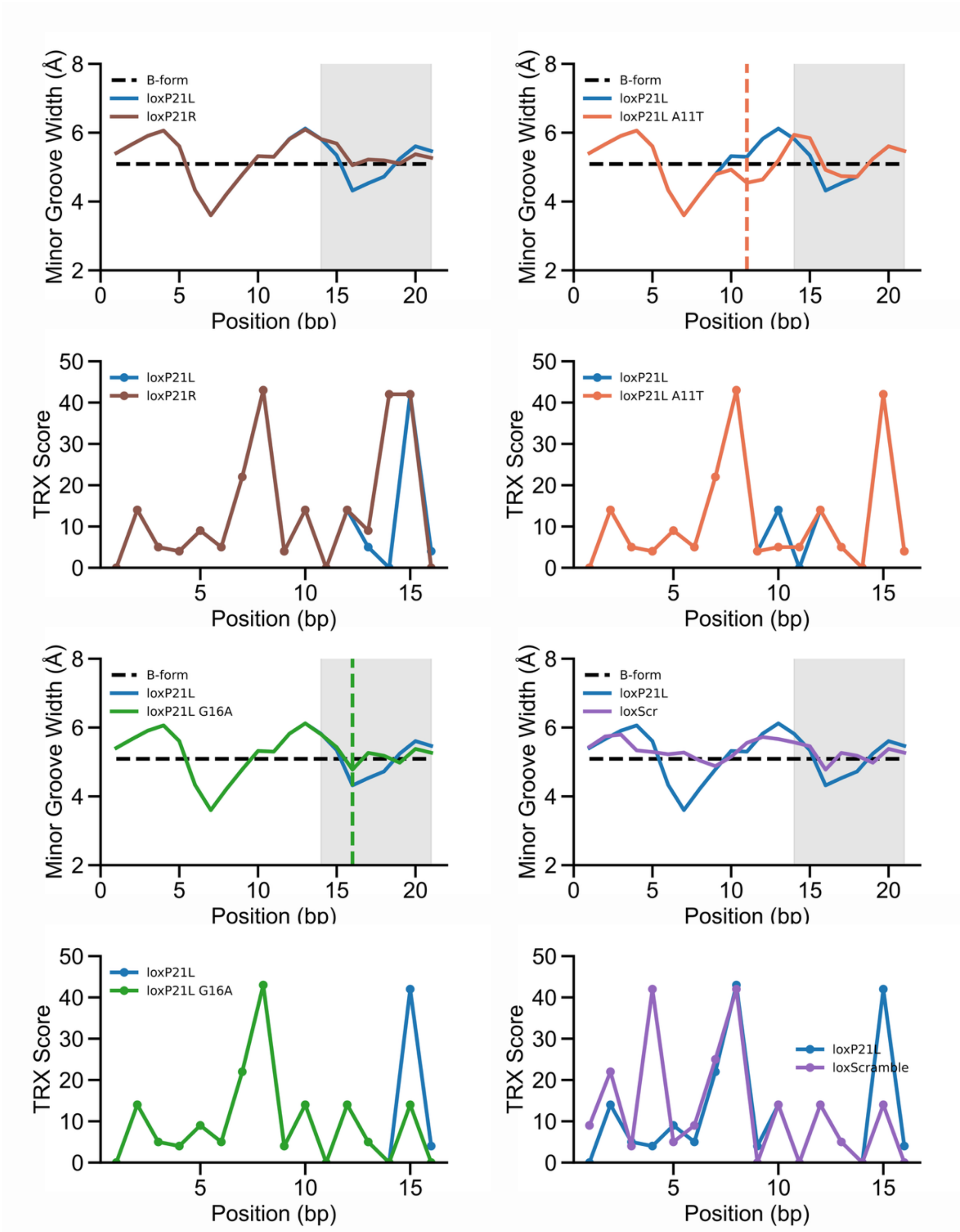

Supplemental Figure 1. Predicted minor groove width and TRX (twist, roll, x-displacement) scores for *loxP* half site variants using the DEEP DNAshape webserver (https://deepdnashape.usc.edu/) and TRX scores from B. Heddi et al., *Nucleic Acids Res* 2010. Shaded regions indicate the position of the *loxP* spacer sequence and vertical dashed lines indicate the position of single point mutations. Black dashed lines indicate minor groove width for B-form DNA. Smaller TRX scores are attributed to more rigid DNA sequences.

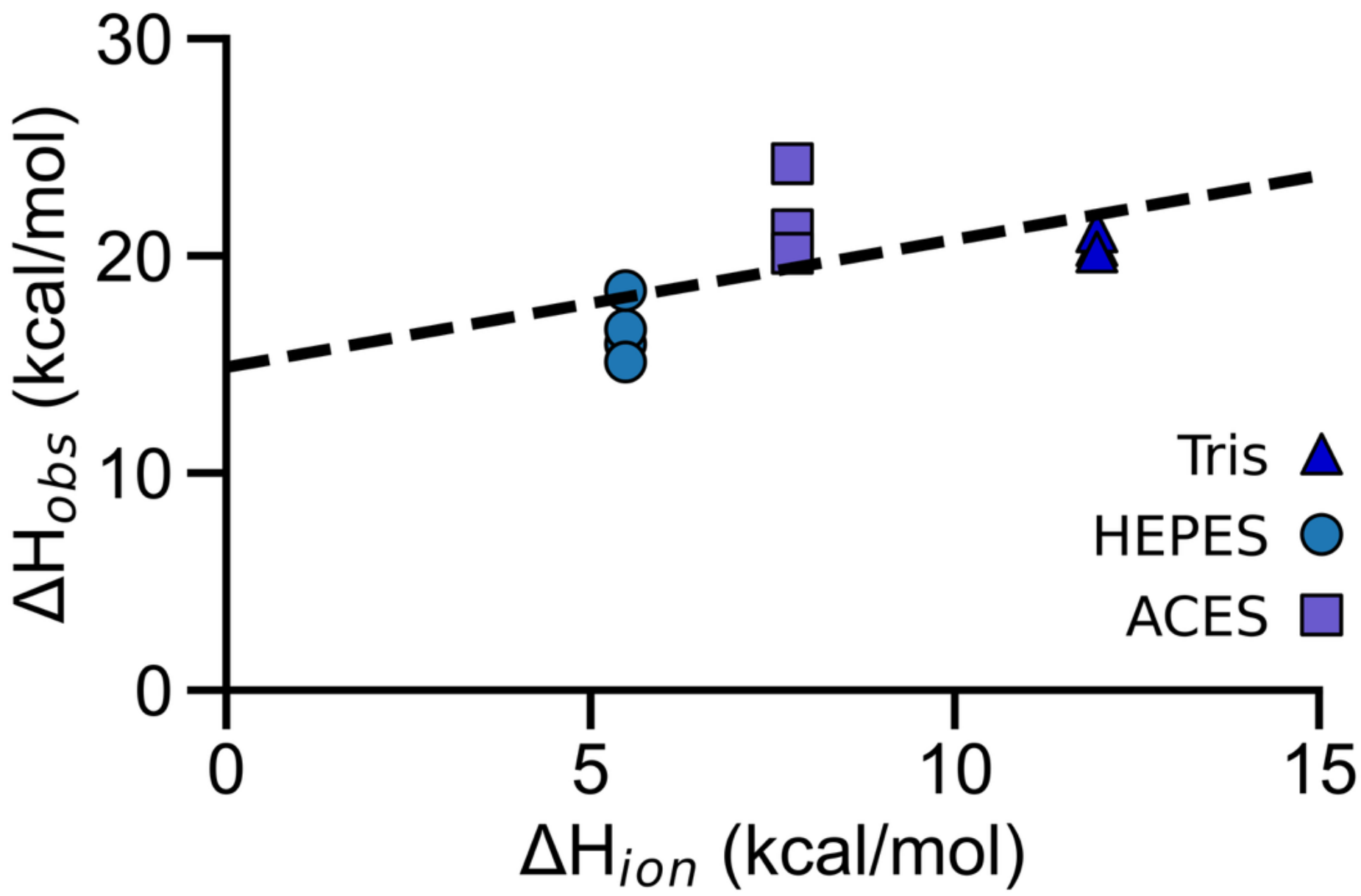

Supplemental Figure 2. Effect of buffer ionization on enthalpy of Cre-loxP half site binding. The observed enthalpy (ΔH_{obs}) of WT Cre binding *loxP*21L substrates was measured at 15 °C in three buffers exhibiting different enthalpies of ionization (ΔH_{ion}). Fitting this relationship yields a buffer corrected enthalpy of binding of +14.86 kcal/mol and 0.6 ± 0.3 ionization events at pH 7.0.

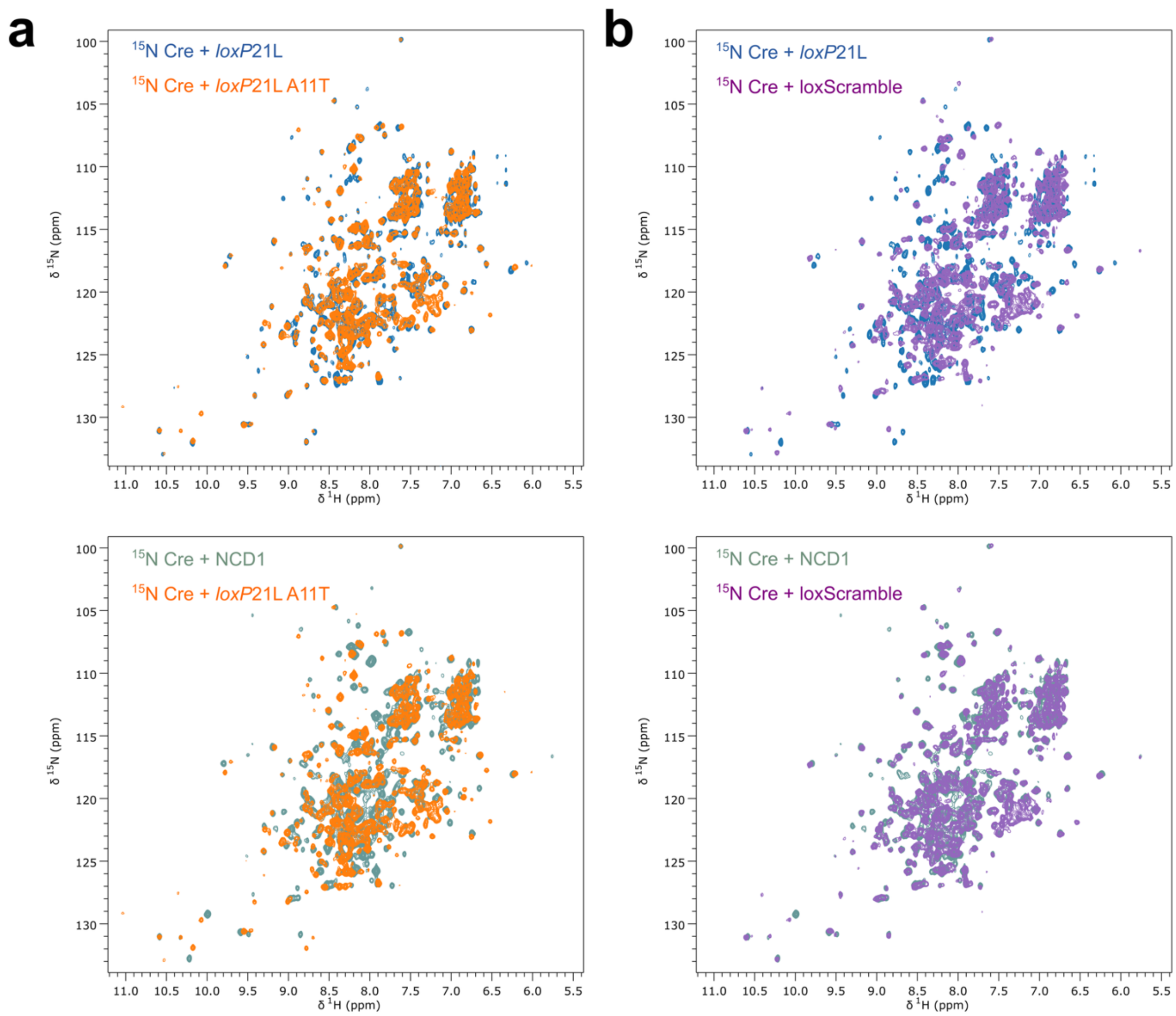

Supplemental Figure 3. TROSY-HSQC spectra of [U-^{15}N] Cre in complex with DNA. Spectra of Cre mixed with excess cognate *loxP*21L (blue) or noncognate DNA (teal) were compared to spectra of Cre mixed with excess loxP21L A11T (orange) or loxScramble (purple). Qualitatively, it appears that the spectrum of Cre-A11T overlays more closely with loxP21L whereas the spectrum of Cre-loxScramble overlays more closely with noncognate DNA.

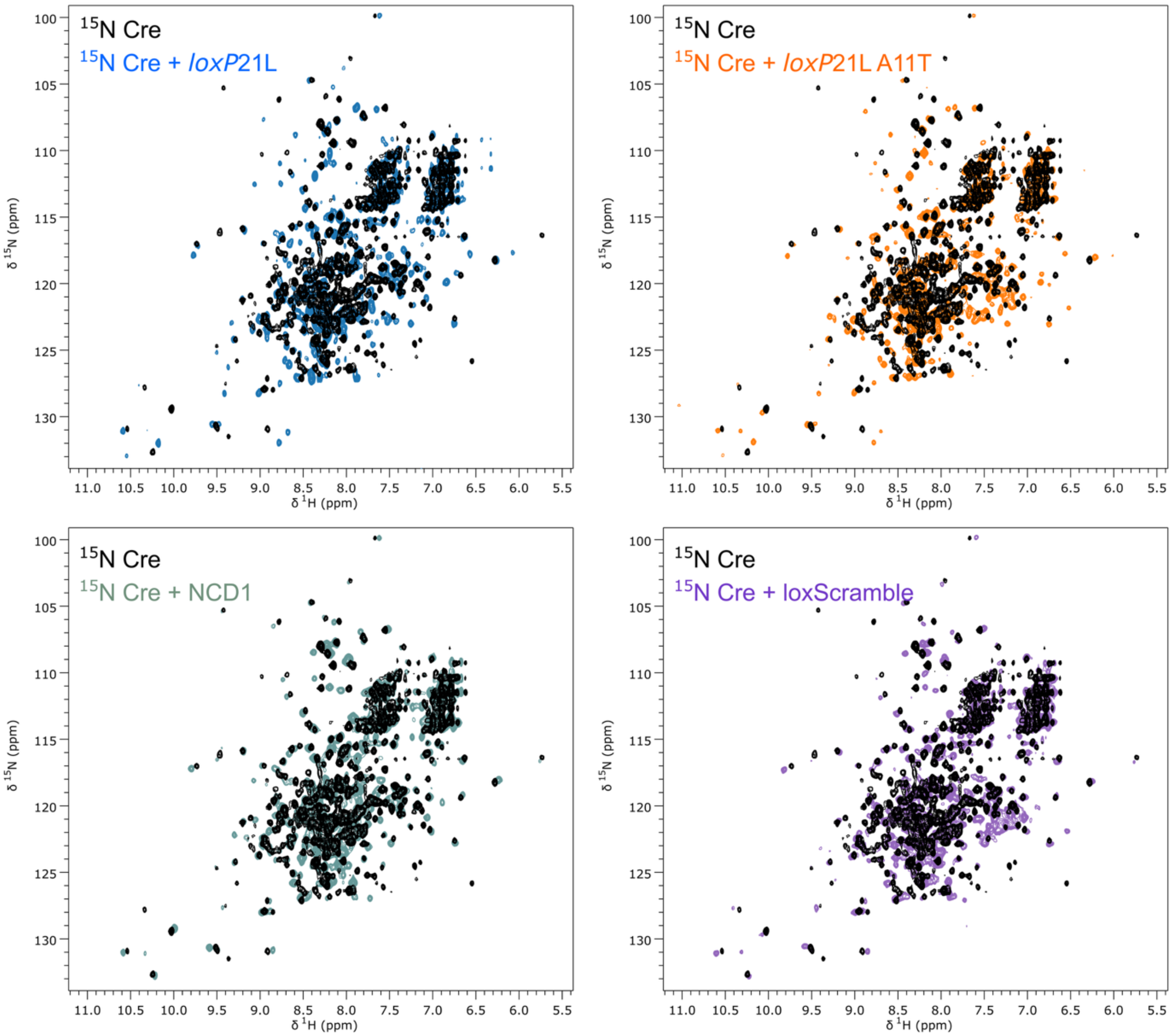

Supplemental Figure 4. TROSY-HSQC spectra of apo [U-^{15}N] Cre compared to Cre mixed with excess DNA. Spectra of ^{15}N Cre (apo, black) was compared to spectra of Cre mixed with excess *loxP*21L (blue), *loxP*21L A11T (orange), noncognate DNA (teal) or loxScramble (purple).

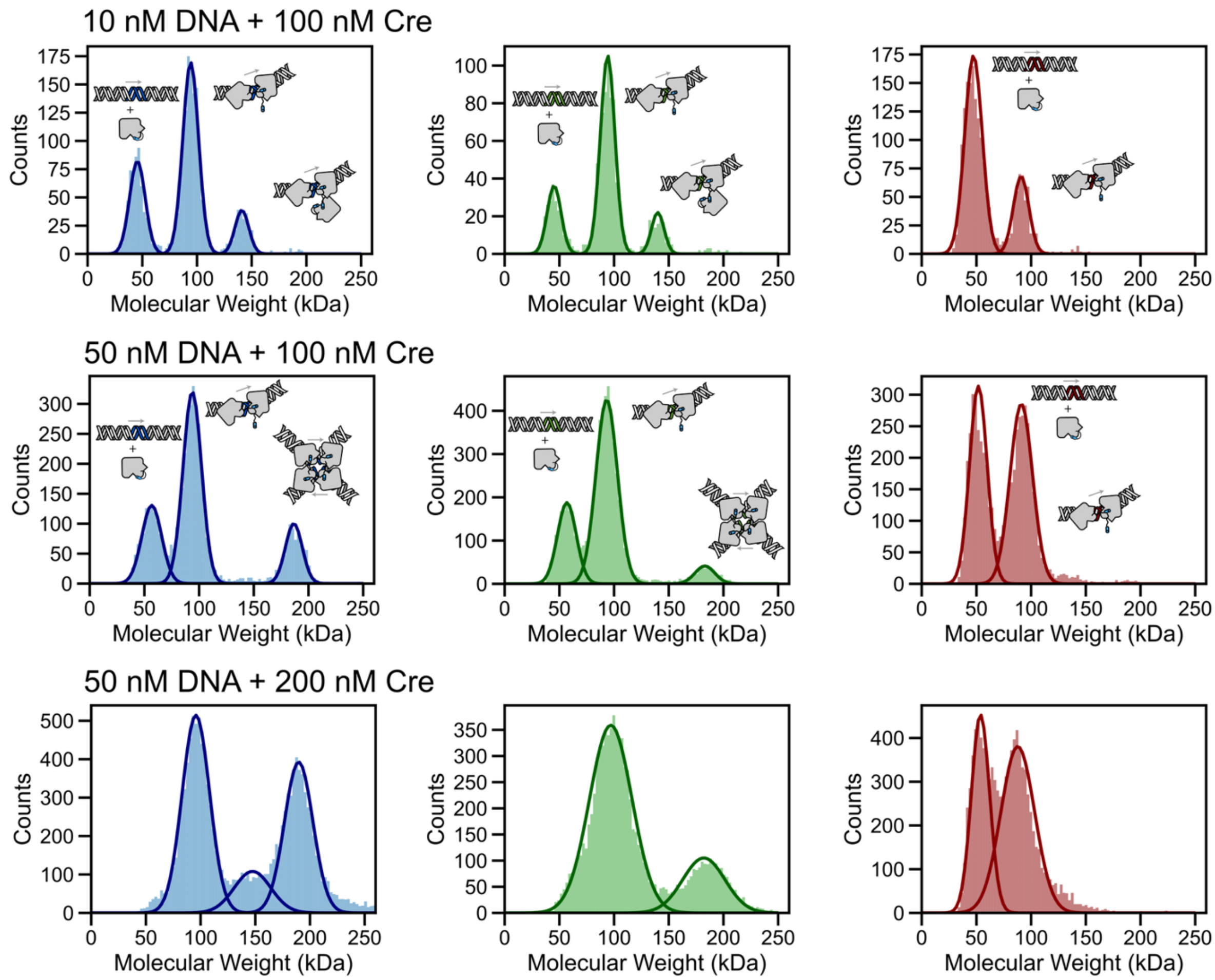

Supplemental Figure 5. Mutations in the loxP spacer perturb synapsis. Population distributions for assembly intermediates of Cre- *loxP* (blue), *loxP* G16A (green), and loxSR (red) complexes at varying protein and DNA concentrations. We observe a ~45 kDa species that corresponds to free Cre or the monomer bound to loxP, a 94 kDA species that corresponds to a Cre2-loxP dimer, a 140 kDa species that corresponds to a Cre3-loxP complex, and a 190 kDa species that corresponds to the tetrameric complex. Populations were fit using a gaussian mixture model.

Name	Sequence	ε_{260} (M^{-1} cm^{-1})	Use
loxP21L	5' – GCA TAA CTT CGT ATA ATG TCG – 3'<br>3' – CGT ATT GAA GCA TAT TAC AGC – 5'	333,780	ITC/NMR
loxP21R	5' – GCA TAA CTT CGT ATA GCA TCG – 3'<br>3' – CGT ATT GAA GCA TAT CGT AGC – 5'	334,427	ITC/NMR
loxP21L A11T	5′ – GCA TAA CTT CGT TTA ATG TCG – 3′<br>3′ – CGT ATT GAA GCA AAT TAC AGC – 5′	330,581	ITC/NMR
loxP21L G16A	5' – GCA TAA CTT CGT ATA ATA TCG – 3'<br>3' – CGT ATT GAA GCA TAT TAT AGC – 5'	341,315	ITC
loxScramble	5' – GCA GAC AAG CAT ATA ATA TCG – 3'<br>3' – CGT CTG TTC GTA TAT TAT AGC – 5'	341,074	ITC/NMR
loxP 38	5′ – GCA TAA CTT CGT ATA ATG TAT GCT ATA CGA AGT TAT CG – 3′<br>3′ – CGT ATT GAA GCA TAT TAC ATA CGA TAT GCT TCA ATA GC – 5′	602,426	MP
loxP 38 G16A	5′ – GCA TAA CTT CGT ATA ATA TAT GCT ATA CGA AGT TAT CG – 3′<br>3′ – CGT ATT GAA GCA TAT TAT ATA CGA TAT GCT TCA ATA GC – 5′	602,409	MP
loxSR	5′ – GGA TAA CTT CGT ATA GCA TAT GCT ATA CGA AGT TAT CC – 3′<br>3′ – CCT ATT GAA GCA TAT CGT ATA CGA TAT GCT TCA ATA GG – 5′	605,082	MP

Supplemental Table 1: DNA oligonucleotides used in these studies. DNA sequence is colored to indicate the RBE (blue) and spacer (red). Extinction coefficients shown were used to determine DNA concentration.

DNA Substrate	Run	[Cell] (μM)	n	+/-	ΔG (kcal/mol)	+/-	ΔH (kcal/mol)	+/-	Kd (nM)	+/-
loxP21L	1	15	1.05	0.004	-10.28	0.15	15.94	0.14	15.80	0.23
loxP21L	2	15	1.07	0.005	-10.01	0.12	16.61	0.15	25.33	0.30
loxP21L	3	10	0.97	0.010	-10.37	0.44	18.40	0.44	13.50	0.38
loxP21L	4	4	1.16	0.010	-10.33	0.33	15.11	0.33	14.48	0.32
		Mean	1.06	0.01	-10.25	0.26	16.52	0.27	17.28	0.31
		Stdev	0.08		0.16		1.40		5.45	

DNA Substrate	Run	[Cell] (μM)	n	+/-	ΔG (kcal/mol)	+/-	ΔH (kcal/mol)	+/-	Kd (nM)	+/-
loxP21L T7A	1	4	1.26	0.02	-10.11	0.18	13.98	0.28	27.06	0.54
loxP21L T7A	2	4.5	1.24	0.01	-9.57	0.11	14.24	0.21	68.06	1.01
loxP21L T7A	3	4	1.34	0.01	-9.69	0.10	14.08	0.18	55.82	0.72
		Mean	1.28	0.01	-9.79	0.13	14.10	0.22	50.31	0.76
		Stdev	0.05		0.28		0.13		21.04	

DNA Substrate	Run	[Cell] (μM)	n	+/-	ΔG (kcal/mol)	+/-	ΔH (kcal/mol)	+/-	Kd (nM)	+/-
loxP21L A11T	1	5	1.16	0.02	-9.04	0.09	10.63	0.16	137.47	1.31
loxP21L A11T	2	5	1.10	0.04	-9.37	1.20	8.71	0.39	77.07	9.87
loxP21L A11T	3	5	1.09	0.01	-8.96	0.08	10.82	0.16	156.74	1.33
		Mean	1.11	0.02	-9.12	0.45	10.05	0.24	123.76	4.17
		Stdev	0.04		0.22		1.17		41.56	

DNA Substrate	Run	[Cell] (μM)	n	+/-	ΔG (kcal/mol)	+/-	ΔH (kcal/mol)	+/-	Kd (nM)	+/-
loxP21L G16A	1	3	1.23	0.01	-10.50	0.16	14.54	0.22	10.73	0.16
loxP21L G16A	2	3	1.20	0.01	-10.59	0.20	15.10	0.26	9.18	0.16
loxP21L G16A	3	3	1.16	0.01	-10.63	0.16	15.19	0.21	8.46	0.11
		Mean	1.20	0.01	-10.57	0.17	14.94	0.23	9.45	0.14
		Stdev	0.03		0.07		0.35		1.16	

DNA Substrate	Run	[Cell] (μM)	n	+/-	ΔG (kcal/mol)	+/-	ΔH (kcal/mol)	+/-	Kd (nM)	+/-
loxScr	1	10	1.12	0.04	-8.06	0.12	4.43	0.14	765.33	11.78
loxScr	2	10	1.18	0.02	-7.99	0.07	5.22	0.09	861.95	7.12
loxScr	3	5	1.03	0.04	-8.42	0.12	5.42	0.17	403.60	5.51
loxScr	4	4.5	1.06	0.04	-8.19	0.13	3.67	0.13	604.43	9.45
		Mean	1.10	0.03	-8.16	0.11	4.68	0.14	658.83	8.47
		Stdev	0.07		0.19		0.80		200.59	

Supplemental Table 2. Thermodynamic parameters obtained from fitting titrations of mutant loxP half site sequences at 15°C. Uncertainties in individual fits were estimated by bootstrapping with 1000 iterations.

Buffer	Run	[Cell] (μM)	n	+/-	ΔG (kcal/mol)	+/-	ΔH (kcal/mol)	+/-	Kd (nM)	+/-
HEPES	1	15	1.05	0.004	-10.28	0.15	15.94	0.14	15.80	0.23
HEPES	2	15	1.07	0.005	-10.01	0.12	16.61	0.15	25.33	0.30
HEPES	3	10	0.97	0.010	-10.37	0.44	18.40	0.44	13.50	0.38
HEPES	4	4	1.16	0.010	-10.33	0.33	15.11	0.33	14.48	0.32
		Mean	1.06	0.01	-10.25	0.26	16.52	0.27	17.28	0.31
		Stdev	0.08		0.16		1.40		5.45	

Buffer	Run	[Cell] (μM)	n	+/-	ΔG (kcal/mol)	+/-	ΔH (kcal/mol)	+/-	Kd (nM)	+/-
Tris	1	4	1.00	0.02	-11.16	2.79	20.47	0.56	3.36	0.84
Tris	2	4	0.95	0.01	-10.85	0.14	21.16	0.26	5.75	0.07
Tris	3	4	0.93	0.06	-11.06	3.87	20.18	2.58	4.00	1.40
		Mean	0.96	0.03	-11.02	2.27	20.60	1.13	4.37	0.77
		Stdev	0.04		0.16		0.51		1.23	

Buffer	Run	[Cell] (μM)	n	+/-	ΔG (kcal/mol)	+/-	ΔH (kcal/mol)	+/-	Kd (nM)	+/-
ACES	1	2.5	1.02	0.02	-11.71	4.00	21.20	0.81	1.28	0.43
ACES	2	2.5	1.01	0.03	-11.07	4.10	24.24	1.05	3.93	1.45
ACES	3	2.5	1.02	0.01	-10.55	0.10	20.11	0.19	9.79	0.10
		Mean	1.02	0.02	-11.11	2.73	21.85	0.68	5.00	0.66
		Stdev	0.01		0.58		2.14		4.35	

Supplemental Table 3. Thermodynamic parameters from fits of Cre-*loxP*21L titrations with varying buffer enthalpy of ionization. Buffers contain 20 mM of buffering agent at pH 7.0 and 250 mM NaCl. Uncertainties in individual fits were estimated by bootstrapping with 1000 iterations.

DNA Substrate	Temperature (K)	n	+/-	ΔG (kcal/mol)	+/-	ΔH (kcal/mol)	+/-	Kd (nM)	+/-
loxP21L	288	1.06	0.08	-10.25	0.16	16.52	1.40	17.28	5.45
loxP21L	293	1.05	0.01	-10.48	0.30	11.25	0.44	15.60	7.60
loxP21L	298	1.19	0.02	-10.79	1.84	8.29	0.45	15.03	11.00
loxP21L A11T	288	1.11	0.04	-9.12	0.21	10.05	1.16	123.76	4.17
loxP21L A11T	293	1.07	0.02	-9.38	0.10	8.12	0.14	100.76	1.06
loxP21L A11T	298	1.11	0.01	-9.05	0.11	7.42	0.46	178.08	33.20
loxScramble	288	1.10	0.07	-8.16	0.19	4.68	0.80	658.83	200.59
loxScramble	293	1.02	0.11	-8.27	0.23	3.98	1.03	717.08	279.60
loxScramble	298	1.02	0.13	-8.38	0.22	3.53	0.72	743.98	251.29

Supplemental Table 4. Thermodynamic parameters from fitting titrations at varying temperatures.

Uncertainties represent the standard deviation of all replicates.

Sample	^{1}H Carrier (ppm)	^{1}H SW (ppm)	^{15}N Carrier (ppm)	^{15}N SW (ppm)	NS	1H TD	15N TD
Apo Cre	4.697	21.8495	117	40	24	2048	256
Cre + loxP21L	4.696	18.0258	117	50	40	2048	280
Cre + loxP21R	4.698	21.8495	116	36	48	2048	256
Cre + NCD1	4.696	18.0258	117	50	40	2048	280
Cre + NCD2	4.697	18.0258	117	50	40	2048	280
Cre + loxP21L A11T	4.698	21.8495	116	36	48	2048	256
Cre + loxScramble	4.697	21.8495	116	36	40	2048	256

Supplemental Table 5: Acquisition parameters for TROSY-HSQC spectra.
